# Research on emotional intelligence among Indian teachers: A Systematic Review and meta-analysis of its correlation with health parameters and impact of gender

**DOI:** 10.12688/f1000research.143151.1

**Published:** 2023-11-28

**Authors:** Mamta Pandey, Deepti Sharma

**Affiliations:** 1 Faculty of Management, Uttaranchal University, Dehradun, Uttarakhand, 248001, India

**Keywords:** Emotional intelligence, teachers, trait EI, education system, personal health, professional health, gender effect on EI

## Abstract

**Background:**

Emotional intelligence is the self-perception related to identification and regulation of emotions. Several studies have been done among Indian teachers evaluating emotional intelligence in relation to demographic, professional and various psychological parameters, but the variety of scales, teacher types, and conflicting results makes it difficult to draw any meaningful conclusions from this heterogeneous data.

The present work aims to synthesize the available data by both qualitative and quantitative analysis and is the first such attempt to include only Indian studies in this field. The main objectives were to determine the correlation of emotional intelligence with teachers’ health parameters and to study the gender difference in emotional intelligence.

**Methods:**

After a thorough literature search in Google, Google scholar, Scopus, Web of science and Pubmed, fifty-five Indian studies were selected which empirically examined teachers’ emotional intelligence, either alone or in association with another parameter evaluating teachers’ psychological health and performance. After qualitative assessment of major findings, quantitative analysis was performed. Three separate meta-analysis were carried out. The first one with fifteen effect sizes among 3291 participants evaluated correlation with personal health parameters. The second with nineteen effect sizes in 4165 participants evaluated correlation with professional health parameters. The third with twenty-six studies involving 6005 participants assessed effect of gender.

**Results:**

The results show that almost all studies have used a trait measure, teachers’ emotional intelligence is positively correlated with both personal and professional health parameters and gender has no effect on emotional intelligence.

**Conclusion:**

Major limitations are a very high degree of heterogeneity of the data, incomplete description of the scales, inadequate randomization and small sample sizes in many studies. The results indicate the importance of emotional intelligence in both personal and professional life of teachers and no effect of gender preparing a solid base for future research.

## Introduction

Emotional intelligence (EI) has gained wide popularity since its inception around three decades back, both among popular media and research community. The emotional aspect of human intelligence is such an attractive concept that practicing psychologists, policymakers, motivational speakers, or business managers, all are drawn towards it and have carried forward the concept to a wider audience and general population. While the actual term is relatively recent, most authorities agree that the concept is very old. If Goleman traces its early origins in the writings of Aristotle ( Goleman, 1995), then Bar-On gives credit to Darwinian theory of evolution for inspiring his research ( Bar-On, 2006). In modern times, Thorndike’s ‘social intelligence’ can be taken as the first scientific enquiry seriously looking into the realm of non-intellective factors ( Thorndike, 1920), which were further elaborated by many later researchers. The landmark studies of Mayer and Salovey in 1990s ( Salovey and Mayer, 1990; Mayer, Salovey and Caruso, 2004), the spectacular success of Goleman’s subsequent book and theoretical research of Bar-On established the solid scientific framework for this construct. The three basic models proposed by these three groups of scholars, along with definitions and measuring tools based on them have been extensively discussed in literature previously. The most important development since the early elaboration of three fundamental models is the distinction between ability EI and trait EI. This distinction is now standard in international scientific literature but less well recognized in Indian studies and needs emphasis. According to Petrides and Furnham (2000a), who highlighted this at the turn of the century, Ability EI tests measure constructs related to an individual’s theoretical understanding of emotions and emotional functioning, whereas Trait EI questionnaires measure typical behaviors in emotion-relevant situations and self-rated perception. The main differentiating feature of this classification is that EI type is best defined by method of measurement: Measures that are based on self-report items are termed “trait EI” whereas measures that are based on maximum performance items are termed “ability EI”. Later research has shown that trait EI is better correlated with psychological, health, organizational or behavioral parameters than ability EI ( Petrides *et al.,* 2016). The term “mixed” is now frequently used in the literature to refer to EI tools that measure a combination of traits, social skills and competencies and overlaps with other personality measures ( O’Connor *et al.,* 2019).

### Indian EI scales

A simple literature search of EI studies in India reveals that there is a plethora of tests developed by Indian scholars. This is both a boon and a bane for EI research in India. While it shows the huge interest shown by Indian scholars for this rapidly developing field as well as the depth of research aptitude and methodology, it also creates a situation where most of these tests are used by very few studies and thus lack the widespread validity and reproducibility required of a rigorous scientific tool. It is hardly surprising that none of these tools have gained wide recognition outside India, as it’s simply not possible to project any one of them as a standard Indian scale. A detailed analysis of these scales, including their strengths and weaknesses and uses in Indian studies is beyond the scope of this review, but a brief introduction of three of the commonly used tests is presented here. The first one is Emotional Competence Scale (ECS) developed by Sharma and Bhardwaj in 1993 with publication of its psychometric properties in 1994 ( Bharadwaj and Sharma, 1994). It seems to be the first such scale in India but now not available online and its conceptual framework seems somewhat different from the later models of emotional intelligence. The two most common Indian scales are Emotional intelligence Scale (EIS) developed by Hyde, Pethe and Dhar ( Hyde and Pethe, 2001) and Emotional Quotient test (EQ test) by Singh and Chaddha ( Singh, 2006). Both were first published around the same time in 2001.

### Hyde test

This is a self-rated test comprising 34 questions. In the manual describing the scale, the authors note that they had come across only two international scales, one by Cooper and Sawaf (1997) and another by Goleman (1995). A lot of questions in this scale pertain to the behaviour or perception of an individual working within an organization and the related issues of motivation, encouragement and decision making etc. Being a self-report measure, it would be classified as measure of trait EI, although like Schutte Self-report Emotional intelligence test or SSEIT ( Schutte *et al.*, 1998), it is primarily based on a definition of EI which is more in sync with the ability model. Being available in both English and Hindi language, it is not surprising that it received widespread acceptance and is one of the most commonly used tests by Indian scholars, both in education and other sectors as well. A brief comparison with the almost similar SSEIT shows that the later test is more generalized in its approach and suitable for different populations while the former is a suitable test mainly for working executives. While SSEIT is more focused on how one ‘feels’, EIS seems more focussed on how one ‘acts’ under certain emotional situations.

### D Singh test

Singh and Chadha developed two different tests which were published in the book by D Singh in 2001, named *Emotional Intelligence at Work: A Professional Guide.* As the name of the book itself suggests, these tests are also more suitable for working professionals, although the authors have tested the validity of the scales in different sample populations. The first scale, called Emotional Quotient test, which is also the more popular and used scale is based on maximum performance with right and wrong answers (ability measure) comprising 22 questions based on emotional situations. The second one, called Emotional Intelligence Scale, comprises 60 questions and is a self-report measure with responses in Likert type scale ranging from strong disagreement to strong agreement (trait measure). Being a lengthy and less rigorously tested measure, it is very rarely used by other scholars. The only relevant reference in the book is of Goleman’s book, and the writing style and practical examples give a clue to the obvious influence of that book, but there is no mention of any other international tests in the book.

It appears that both these groups had not come across the three other important and later on more widely used tests developed around the same time, the EQ-i by Bar-On in 1997, the SSEIT by Nicola Schutte and others in 1998 and the MEIS by Mayer, Caruso and Salovey in 1999, which was the precursor of MSCEIT (Mayer Salovey Caruso Emotional Intelligence Emotional Intelligence test) ( Ackley, 2016). In this respect, the two Indian scales assume more importance as it was a big scholarly feat.

### Previous meta-analyses on EI

Starting with the landmark meta-analysis by Van Rooy and Viswesvaran (2004), which not only examined the psychological bearing of EI and similarity and variance with cognitive intelligence and personality but also assessed its predictive role with various life outcomes, there have been several attempts by various scholars to present the ever-increasing data in a systematic way. Schutte *et al.* (2007) established that EI had a positive correlation with mental, psychosomatic and physical health and trait measures had better correlation than ability measures. Martins *et al.* (2010) carried forward this research and showed that EI is better correlated with mental and psychosomatic health than physical health and TEIQue showed strongest association among all trait measures. Joseph and Newman (2010) proposed a cascading model of EI and tried to integrate ability and mixed models. They also looked at gender difference in EI scores, which has been a subject of many studies with contrasting results ( Van Rooy *et al.*, 2006), and showed that while women fared better than men in ability measures, for self-report measures, there was no gender difference. In recent years, many meta-analyses have been published linking EI with students’ academic performance ( Maccann *et al.*, 2019; Sánchez-Álvarez *et al.*, 2020) life satisfaction ( Hartanto and Helmi, 2021), and adolescents’ subjective well- being ( Llamas-Díaz *et al.*, 2022). To the best of our knowledge, only two meta-analyses have been conducted by Indian researchers so far in EI domain. Shukla and Srivastava (2016) examined the association of EI with demographic and occupational parameters like job stress and satisfaction, while Manimozhi and Srinivasan (2018) assessed the correlation of EI with students’ academic achievement. Both of them included some Indian studies. There has been a lot of research among Indian teachers with respect to their EI and its relation with demographic, personal and professional attributes but no meta-analysis has been attempted to synthesize their results and present their findings in a coherent manner. This systematic review and meta-analysis is the first attempt in this direction with inclusion of only Indian studies in this field, and aims to fill an important research gap regarding the impact of teachers’ EI on their health and performance.

### Research gap

A lot of studies and subsequent reviews and meta-analyses have examined the effect of EI on students’ personal and professional metrics, but no such work has been done regarding Indian teachers. Individual studies have reported conflicting results regarding the association of EI with factors like teacher effectiveness, job satisfaction or mental health and the role of age, professional qualification or gender. The inceasing body of research in this field now needs a systematic collation and synthesis of available data to gain insightful knowledge from this seemingly conflicting literature. The role of gender on EI is one such issue which needs to be explored in both large scale studies as well as systematic meta-analysis to reconcile the conceptual framework with solid evidence. Studies in other sectors, both in India and abroad have also reported similarly conflicting results regarding gender ( Bar-On *et al.*, 2000; Petrides and Furnham, 2000b; Extremera *et al.*, 2006; Van Rooy *et al.*, 2006; Brackett *et al.*, 2006; Malouff and Bhullar, 2009; Fernández-Berrocal *et al.*, 2012; Pooja and Kumar, 2016; Meshkat and Nejati, 2017). The impat of EI on teachers’ performance and health also needs emphasizing so that policy makers and school administrators become aware regarding the needs and interventions in this regard.

**Study objectives**

This review paper was written primarily with two objectives. First, to collect and collate all available Indian studies on emotional intelligence done among teachers and to analyze the data qualitatively and present a systematic synthesis of major findings. Second, where available and feasible, quantitative meta-analysis would be attempted to gain more insight into the research done in this field. PRISMA guidelines were followed for screening and inclusion of studies for systematic review as well as for presenting the major findings of meta-analysis. The PRISMA checklist for abstract and review (2020) for the present work is available as underlying data ( Pandey, 2023), with details provided at the end of this paper. Similarly, SAGER guidelines were followed for reporting and analyzing all relevant data regarding gender. The main focus for quantitative analysis was on the impact of demographic and professional attributes on EI and teachers’ personal and professional parameters associated with EI. The main objectives are listed as follows:

1. To collect, tabulate and analyze all available studies of EI done among Indian teachers in the last decade.
2. To study the relationship of demographic and professional factors like age, gender, educational qualification and level of teaching on EI and perform meta-analysis of available studies.
3. To study the relationship of EI with other parameters concerned with personal and professional health of teachers.

**Hypotheses**

Based on the literature review and objectives of the present study, the following hypotheses were formed.

H01. Emotional intelligence of teachers has no relation with personal and professional health parameters.

H1 (alternative). Emotional intelligence of teachers is positively correlated with personal and professional health outcomes.

It is well established that EI is positively correlated with various professional outcomes like students’ academic performance and mental and psychological health. Organizational parameters like job satisfaction, performance, occupational stress, have also been associated with EI. Teacher’s EI can affect both their personal and professional life and can have far reaching consequences on the life of students as well. We propose that EI of teachers will have a significant correlation with parameters associated with both personal and professional attributes like teaching effectiveness and attitude, job satisfaction, stress, burnout, and mental health etc.

H02. There is no effect of age, gender and educational qualification on teachers’ emotional intelligence.

H2 (alternative). There is an effect of age, gender and educational qualification in teachers’ emotional intelligence.

The conceptual definition of trait EI places it near personality and quite distinct from cognitive intelligence. As such, it should not change with age, experience or educational qualification. Regarding gender, although women are thought to be better at handling and expressing emotions, the studies with different EI measures have reported contrasting results. As this is the first meta-analysis of Indian studies among teachers with different scales, we make the null hypothesis that gender should not affect EI.

## Methods

The following eligibility criteria and search strategies were used for screening and inclusion of studies.

### Identification and search

All studies available in English language on Google, Google Scholar, PubMed, Scopus and WOS databases, done among Indian teachers published between 2009 and 2022 were screened for the review. The keywords used were “India, Indian, emotional intelligence, trait EI, ability EI, teachers, educators, primary/secondary/college teachers, professional institutes, education system and educational institutes”. Both the authors independently searched and screened available papers and met at regular intervals to exclude duplicate papers.

### Inclusion criteria for systematic review

(1) Original, empirical studies which measured EI of teachers, either alone or in association with other parameters.
(2) The year 2009 was taken as a cut-off period to make data more relevant and current as initial screening revealed first studies from that year.

### Inclusion criteria for meta-analysis

(1) Studies which reported the following parameters and conducted relevant statistical analysis: age, gender, educational qualification.
(2) Studies which conducted a correlation analysis between EI and a second parameter associated with psychological heath or occupation.

### Screening and eligibility

After initial screening of papers, subsequent papers were searched in the references of those papers. All efforts were taken to collect as many studies as possible. Both the authors screened all abstracts and for those found suitable, full papers were retrieved. Duplicate papers were carefully excluded. All relevant papers were included for qualitative analysis and out of those, after further screening, only certain papers with relevant statistical analysis were included for quantitative meta-analysis. To reduce the chances of missing studies due to publication bias, all the references in a given paper were screened for any relevant studies and carefully assessed. Despite that, some of the studies might have been missed, and studies in print only journals with no full text available online were not included.

### Coding of studies

Studies were coded for type of teachers used as sample population (trainee teachers, school teachers, and college teachers), type of EI scale used (ability, trait, mixed), demographics (age and gender), educational qualification, and teachers’ personal or professional attributes (second parameter in relation to EI). All such parameters were classified as personal or professional attributes depending upon whether they were primarily concerned with teachers as individuals (e.g. personality, stress, life satisfaction, spiritual intelligence) or professionals (e.g. occupational stress, job satisfaction, teacher efficiency and attitude etc.). This was done both for presenting the seemingly unrelated datasets from different studies in a coherent and relatable way and for the purpose of meta-analysis. Like previous work in this field, categorization of different parameters into broad categories helps in building proper evidence and can be used by future researchers to plan their studies in a more systematic way. Both the authors independently coded the studies and any difference was resolved after discussion.

### Statistical analysis

Meta-essentials, a set of free workbooks available online with Excel sheets was used for all statistical analysis ( Suurmond *et al.*, 2017). There are standard formats for different types of analysis in this software with readymade excel sheets which require only the correct input at various places and changes in settings as per the requirement of the meta-analysis. For correlation coefficients, it uses Fisher’s r to z transformation for calculation of combined effect size. This avoids the skewness of correlation coefficient and it can be assumed to be normally distributed so that tests of significance can be applied in a meaningful way. Data are presented as summary statistics, meta-analysis with combined effect size calculation, heterogeneity data, Forest plot for graphical representation of effect sizes, subgroup analysis where applicable and publication bias in the form of funnel plot and Egger’s regression. For subgroup analysis, due to the small number of studies, the ‘tau pooled over subgroup’ setting was used. This is done to avoid calculating an imprecise tau square within subgroups as is likely the case when the number of studies is small. For calculation of effect size in meta-analysis of gender effect on EI, the software automatically calculates Hedges’ g for effect size calculation which accounts for the small sample bias which is seen with Cohen’s d. A random effect model was used for calculation of effect size. Although all the studies have been done in India and all use teachers as subjects, there are still differences in the level of educational institutes (from primary level to college/university/professional level), type of institute (government versus private sector), demographics, educational qualifications, sample size and method of sampling (randomized or convenient). Moreover, the various tests used to measure EI and other parameters add to the heterogeneity of the data. Therefore, fixed effect model was not used.

***Publication bias analysis***

Bias due to non-publication of studies with either negative or insignificant results makes the conclusion of any systematic review and meta-analysis less robust. Several methods to study publication bias are available which constitute an essential part of all systematic reviews. We have evaluated this bias with the help of two most used tools for this purpose. The first one is a funnel plot analysis of all studies used for meta-analysis, which simply plots effect size against either sample size or standard error. The presumption being that if bias is present, the plot is skewed towards right and shows a linear, rather than a funnel shape. The second tool is Egger regression analysis with calculation of regression intercept (zero in case of no bias) with p values.

***Sensitivity analysis***

Sensitivity analysis was intended for two possible scenarios to look for the robustness of the results. The first one with removal of studies with very large effect sizes as these may skew the results and are often a common cause of selection bias. The second sensitivity analysis would be carried out with removal of studies with smaller sample sizes. The purpose of carrying out sensitivity analysis is to see whether altering the methodology or study characteristics changes the overall result of the meta-analysis.

Although no formal tool would be used for critical appraisal or of risk of bias assessment and certainty of evidence as all the studies intended for review are supposed to be observational in design with no intervention, a subjective assessment of methodology would be done to evaluate whether the studies used randomization and how.

## Results

**Figure 1.**
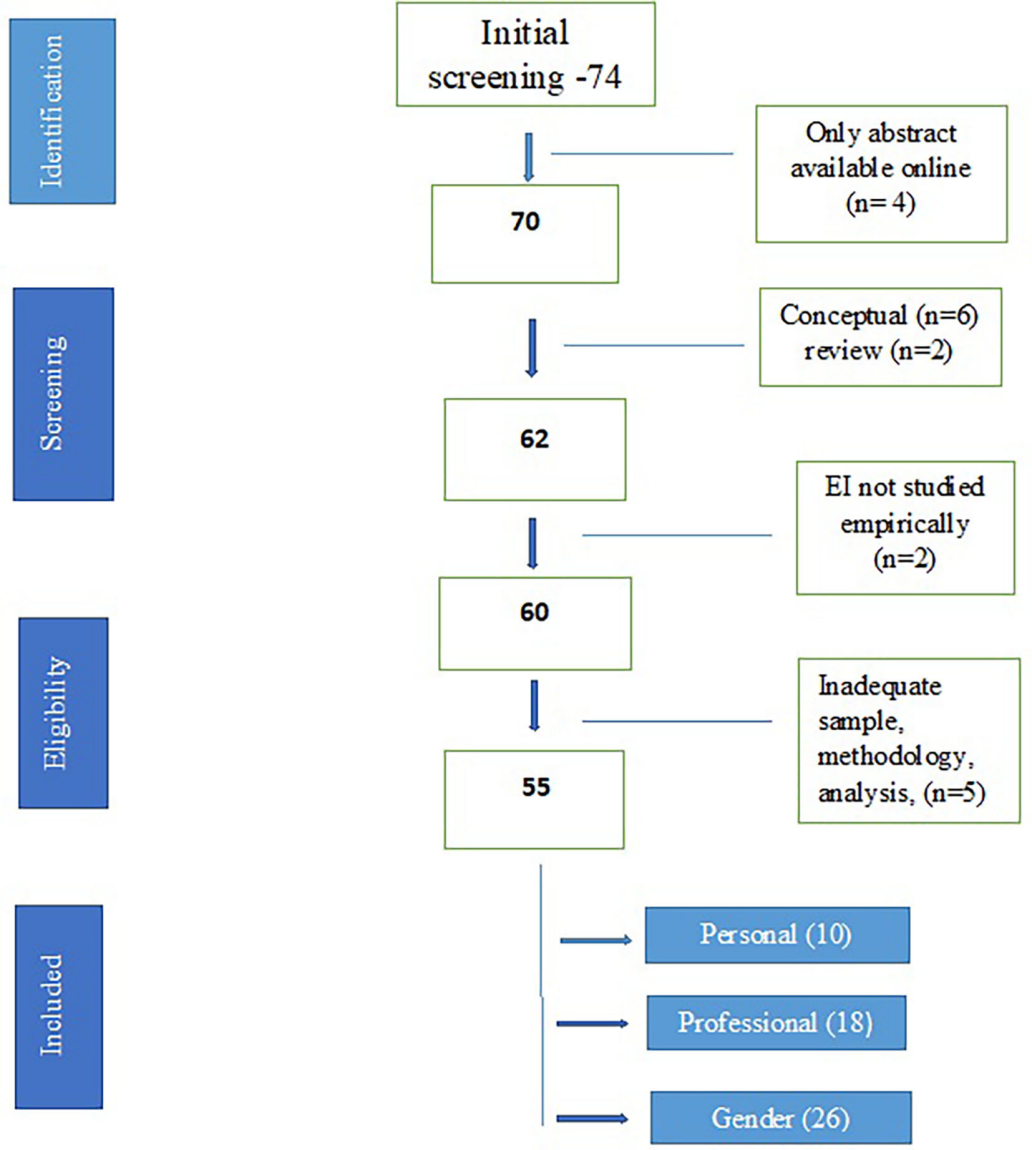
Schematic flowchart for screening of papers.

A total of seventy-four studies were identified through the initial literature search. For four studies, only abstracts were available, two were review articles and six papers were conceptual. Two papers had emotional intelligence in the title but it was not the exact parameter studied empirically through a specific tool. Five studies were excluded due to either a very small sample size, or incomplete details about methodology, measuring scales, statistical analysis or final results. The remaining fifty-five studies were included for qualitative analysis. For the purpose of meta-analysis, studies were further grouped on the basis of adequate data regarding impact of age, gender and educational qualification on EI and correlation with another parameter. Ten studies had adequate data for studying correlation of EI with personal health parameters, which were included in first meta-analysis. Eighteen studies had adequate data for studying correlation of EI with professional health parameters, which were included for the second meta-analysis. Thirty-three studies evaluated gender difference in EI, out of which seven did not have an adequate data set. The remaining twenty-six studies were included in the third meta-analysis. Only six studies examined the relationship of EI with age with only four having adequate data. Similarly, there were only seven studies which evaluated the impact of educational qualification on EI and out of them, only five studies had adequate data for analysis. Therefore, no meta-analysis was conducted for age and educational qualification. Figure 1 depicts the process of inclusion and Table 1 presents the summary characteristics of all the studies.

**Table 1.** Summary statistics for all studies. Studies with results in italics could not be included in meta-analysis due to inadequate data.

Study	N	Teacher type	EI measure		Teachers’ attributes		r	Gender	Age/Edu
			Type	Scale	Category	Parameter			
Mohammadyfar *et al.*, 2009	250	School	Trait	Singh	Pers	OS	-.32		
Lenka *et al.*, 2012	120	School	Trait	Hyde *et al.*	Prof	PD	.66	M>F	
Mandip *et al.*, 2012	245	College	Trait	Wong and Law	Prof	JS	.12		
Jha and Singh, 2012	250	College	Trait	Hyde	Prof	TE	.65/.56	NS	
Mishra and Laskar, 2013	120	School	Trait	Mangal				NS	
Choudhary and Choudhary, 2013	120	School	Trait	Hyde *et al.*	Prof	TE	.43	F>M	
Nisha and Budhisagar, 2013	100	School	Trait	Hyde *et al*.				NS	
Toor, 2013	850	School	Trait	Hyde *et al*.	Pers	*SoI*		*M>F*	
Kant and Lenka, 2013	200	Trainee	Trait	Hyde *et al*.				NS	
Holeyannavar and Itagi, 2013	105	School	Trait	Bhardwaj and Sharma	Pers	MH	-.32		Age
Surana and Rawat *,* 2014	477	Trainee	Trait	Mangal and Mangal				M>F	
Kant, 2014	200	School	?	Kumar G	Pers	*P*		NS	
Kauts and Kaur, 2015	648	Trainee	Trait	Hyde *et al.*	Prof	*TE*			
Begum and Khan, 2015	300	Trainee	Ability	Roqan	Prof	TA	.44	NS	
Maheshwari and Balamulu, 2015	100	School	Trait	Clyde Winters				M>F	
Devi and Babu, 2015	240	College	Trait	?					
Charankumar, 2015	492	School	Trait	Hyde *et al*.				NS	Age/Edu
Mehta, 2015	300	School	Trait	Mehta and Singh	Pers	*P*		NS	
Paul and Thavaraj, 2015	120	School	Trait	Thavaraj				*NS*	*Age/Edu*
Kumar R, 2016	160	College	Trait	Khera *et al.*	Prof	TE	-.11/.19		
Singh and Kumar, 2016	300	School	?	Self-made	Prof	JS	.57		
Das, 2016	100	Trainee	Trait	Self-made	Pers	GI/SpI	.21/.05	NS	
Gitika, 2016	600	School	Trait	Srinivasan e *t al.*					
Sripal and Paramasivam, 2016	100	?School	?	Dwivedi					
Sharma *et al.,* 2016	500	College	Trait	Self-made	Prof	JS			
Rani and Prasad, 2016	63	College	Trait	Self-made	Prof	ATW	.82		
Chaturvedi and Mishra, 2017	300	College	Trait	Hyde *et al.*	Prof	JS	.26	NS	
Anjum and Swathi, 2017	60	School	Trait	SSEIT	Prof	OS	-1.0		
Kaur, 2017	150	School	Trait	Mangal	Pers	LS	.79	NS	
Sharma, 2017	530	School	Trait	Hyde *et al.*	Pers	(M)A	-.16		
Ponmozhi and Ezhilbharathy, 2017	150	School	Trait	Hyde *et al.*				*S*	Age/Edu
Geetha and Sripirabaa, 2017	300	College	Trait	TEI-Que-SF	Pers+Prof	SE, JS	.29/.33	*S*	
Reddy, 2017	332	College	?	Self-made				F>M	Edu
Gill and Sankulkar, 2017	214	?School	Trait	Practical EQ					
Rawat, 2017	477	Trainee	Trait	Mangal and Mangal	Pers	SA	M		
Garg and Islam, 2018	200	School	Ability	Roqan	Prof	TE	.04	NS	
Singh and Singh, 2018	200	Trainee	Trait	Hyde *et al.*				NS	
Rajam Jeya Devi and Kumar, 2018	200	School	Ability	D Singh	Prof	JS	.22		
Suresh and Srinivasan, 2018	152	School	Trait	Srinivasan e *t al.*	Pers	GI/MH	.47/.98	NS	
Toor and Kang, 2018	240	College	Trait	Hyde *et al.*	Prof	OS		NS	
Pugazhenthi and Srinivasan, 2018	200	Trainee	Trait	Mangal and Mangal	Prof	Te		F>M	
Soanes and Sungoh, 2019	352	School	Trait	Mangal	Prof	TE	.18	F>M	Edu
Gopinath, 2020	175	College	Mixed	Bar-On	Pers	SA	.79	*NS*	*Age/Edu*
Uniyal and Rawat, 2020	120	School	Trait	Bhardwaj and Sharma				*NS*	
Chakravorty and Singh, 2020	713	School	Trait	Wong and Law	Pers+Prof	B,WIF, FIW	M		
Sandhu and Agrawal, 2020	400	School	Trait	Khera *et al.*	Pers+Prof	TE,P		M>F	
Nagaraj and Ramesh, 2020	102	School	Trait	Leadership Toolkit				?	
Shanthini *et al.*, 2020	472	School	Trait	Wong and Law					
Mohammad, 2020	221	School	Trait	Sungouh	Prof	JS	.04		
Kamboj and Garg, 2021	200	School	Trait	SSEIT	Pers	MF,Per,SR PW	.58,.41 .46,.33	F>M	
Kant and Shanker, 2021	200	College	Trait	Weisinger	Prof	B	-.22	NS	
Kumara, 2021	200	College	Trait	Hyde *et al.*	Prof	OS	-.46	NS	Age/Edu
Hulinaykar *et al.*, 2021	87	College	Trait	Leadership Toolkit					
Dey and Roy, 2022	608	School	Trait	Self-made					
Pradhan. RK *et al.*, 2022	287	College	Trait	SSEIT	Pers+Prof	WS,EE	.39,.60		

Abbreviations: ATW – attitude towards work, B – burnout, Edu – educational qualification, EE – employee engagement, F – female, FIW – family interference with work, GI – general intelligence, JS – job satisfaction, LS – life satisfaction, (M)A – maladjustment, MF – meaningfulness, M – male, MH – mental health, NS – non-significant, OS – occupational stress, P – personality, Per – Perseverance, Pers – personal, Prof – professional, PD – professional development, PW – Psychological well-being, S – significant, SA – self-actualization, SE – self-efficacy, SoI – social intelligence, SpI – spiritual intelligence, SR – self-reliance, SSEIT – Schutte Self-report Emotional Intelligence Test, Te – teaching efficiency, TE – teacher effectiveness, WIF – work interference with family, WS – workplace spirituality.

### Level and type of school/educational institute

From primary level to professional institutes, different studies have used educators from different level of teaching. Maximum studies (12) have been done on secondary school teachers. Although B.Ed. students are technically not teachers, a lot of studies (10) have been done among them considering them as trainee teachers. Notably, only 5 studies have taken mixed participants from two or more type of institutes. Among government and private sector, 17 studies have taken participants from both sectors while 6 studies each took participants from only one sector. The remaining 30 have not specified this.

***Tests of emotional intelligence (Can change to same font as prev heading)***

There is a lot of heterogeneity in the measurement tool used to assess emotional intelligence. The most commonly used scale is the indigenous scale developed by Hyde, Pethe and Dhar (14 studies with actual mean scores reported in 11) followed by the Teacher’s emotional intelligence inventory developed by Mangal and Mangal (7 studies). Only 12 studies have used international scales with 3 using Wong and Law scale (WLEIS, Law, Wong and Song, 2004)), 3 using SSEIT, while 1 each using Trait EI questionnaire-Short form (TEIQue-SF), Bar-On’s Emotional quotient test, Boyatzis and Goleman’s modified EI test, Clyde Winter EI test, Weisinger Emotional Intelligence Inventory and Leadership toolkit EI scale, although the exact version of Bar-On and Goleman scales have not been specified and some of the papers have not listed the exact reference for these tools.

Among the studies utilizing the scale by Hyde *et al.*, the majority of the participants had a moderate to high EI score with a range of 113.53-150.39. Compared to the mean scores given by the developers of the scale (68, Hyde and Pethe, 2001), the scores in these studies are much higher, almost double in all studies. This can be interpreted in two ways. First, the EI of teachers, as measured by this scale, is very high as compared to general population. Or, given the much larger total sample (the developers used 200 subjects), the mean EI scores found in these studies should be considered “normal” and may be set as new reference point, as the developers have also suggested. That the teachers, as a profession, possess high EI has been seen in other studies, but more population-based studies using the same scale in different professionals can truly prove this point convincingly.

### Risk of bias in included studies

One of the biggest limitations in the majority of the studies is a lack of description of sample collection. Only a handful of studies have used randomized sampling and the rest have either used a purposive convenient sampling or not described the sampling method. Those which have used randomized sampling have not described in detail how the randomization was done.

### Other parameters

Thirty-six studies evaluated another parameter along with EI (in some cases more than one parameter), either in relation to EI or independently. Twelve were categorized as concerning personal health, twenty-four were categorized as concerning professional health and four studies had both. The most common parameter studied is teacher effectiveness (8 studies), closely followed by job satisfaction (7). The other common parameters studied in relation to EI are occupational stress (4) personality (3), self-actualization (2), burnout (2), mental health (2), and general intelligence (2), etc. Most of these parameters had a significant correlation with EI, but not uniformly. Among the eight studies on teacher effectiveness, only 4 had a clear significant, positive correlation with EI, 2 had no correlation, and in three, results were not clear. Among the seven studies on job satisfaction, 4 had a significant positive correlation with EI, one had correlation with some aspects only, one had no correlation and in one study the results were not clearly described. All three studies on self-actualization showed a positive correlation with EI and all 3 studies on occupational stress reported a negative correlation with EI.

Twenty-eight studies assessed the correlation of EI with another parameter with Pearson correlation coefficient, sometimes with more than one parameter or subgroup analysis resulting in multiple r values from a single study. In studies which calculated correlation of EI with multiple parameters, all effect sizes were included. For those studies which did the correlation with either subgroups (gender, type of teachers), or components of EI, or used two different scales for the same parameter, all effect sizes were averaged to avoid giving undue weightage to one sample and study. Some studies assessed another parameter with EI but did not conduct the correlation analysis or used another statistical analysis than Pearson r and therefore were not included in meta-analysis. Some of the studies could not be included either due to different statistical tools used or using component measures which could not be averaged due to opposing nature of parameter studied or multiple subcomponents.

### Subgroups

Subgroup analysis was intended for type of teachers (trainee, school or college) and type of EI scale (ability, trait, mixed) but as almost all studies used trait measure for EI, subgroup analysis was carried out for teacher type only.

### Meta-analysis

Three different meta-analysis were carried out. The initial two for evaluating correlation of EI with personal and professional health parameters and the third for effect of gender on EI.

### Meta-analysis of EI and personal health of teachers

From ten studies examining the correlation of EI with parameters associated with personal health, fifteen effect sizes in 3291 participants were deduced and used for meta-analysis. Table 2 lists all the correlations with confidence intervals and relative weights. Table 3 shows the combined effect size calculated from random effect model and also gives heterogeneity data.

**Table 2.** Correlation of EI with personal health parameters.

Study name	Correlation	CI Lower limit	CI Upper limit	Weight
Mohammadyfar *et al.*, 2009	-0.32	-0.43	-0.20	6.70%
Holeyannavar and Itagi, 2013	-0.32	-0.48	-0.13	6.60%
Das, 2016	0.21	0.01	0.39	6.59%
Das, 2016	0.05	-0.15	0.25	6.59%
Kaur, 2017	0.79	0.72	0.84	6.65%
Sharma, 2017	-0.16	-0.24	-0.08	6.74%
Geetha and Sripirabaa, 2017	0.29	0.18	0.39	6.71%
Rawat, 2017	0.57	0.51	0.63	6.73%
Suresh and Srinivasan, 2018	0.47	0.33	0.59	6.65%
Suresh and Srinivasan, 2018	0.98	0.97	0.99	6.65%
Gopinath, 2020	0.79	0.73	0.84	6.67%
Kamboj and Garg, 2021	0.58	0.48	0.67	6.68%
Kamboj and Garg, 2021	0.41	0.29	0.52	6.68%
Kamboj and Garg, 2021	0.46	0.34	0.56	6.68%
Kamboj and Garg, 2021	0.33	0.20	0.45	6.68%

**Table 3.** Meta-analysis 1 with combined effect and heterogeneity.

**Meta-analysis model**		**Number of incl. subjects**	3291
		**Number of incl. studies**	15
Model	Random effect model	Z-value	2.85
		One-tailed p-value	0.002
Confidence level	95%	Two-tailed p-value	0.004
**Combined effect size**		**Heterogeneity**	
**Correlation**	0.45	Q	1097.47
Confidence interval LL	0.12	p _Q_	0.000
Confidence interval UL	0.69	I ^2^	98.72%
Prediction interval LL	-0.70	T ^2^ (z)	0.37
Prediction interval UL	0.95	T (z)	0.61

**Figure 2.**
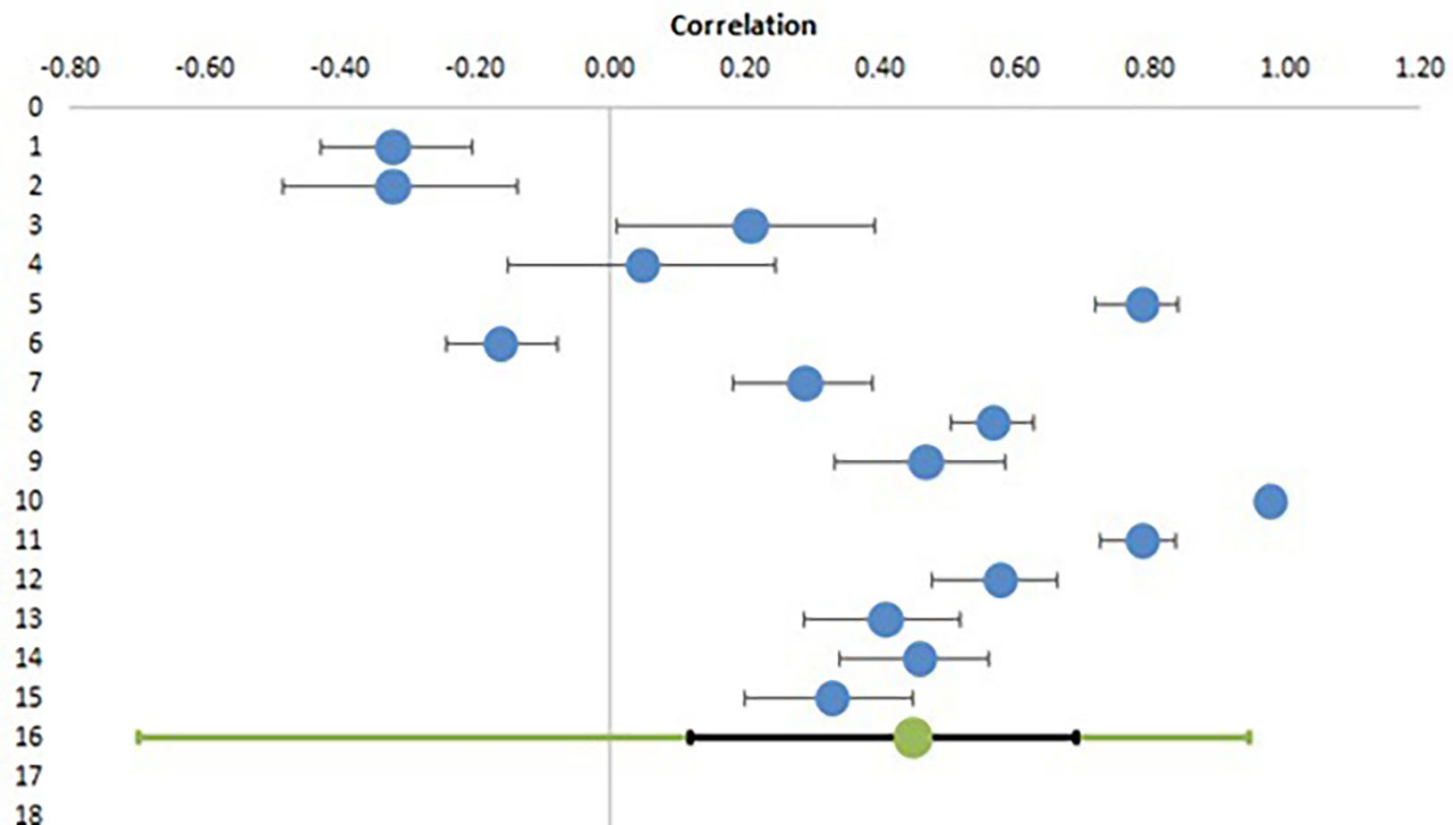
Forest plot for meta-analysis of correlation of EI with personal health.

Figure 2 depicts the Forest plot for the same analysis with individual studies shown in blue circles with their calculated confidence interval and the bottom line showing combined effect size in green with confidence interval in black line and prediction interval in green colour.

It is evident from this meta-analysis that there is a large and significant correlation of EI with personal health of teachers with a combined effect size of 0.45 (95% CI, 0.12 to 0.69). The confidence interval, z value and p values (<0.05) all suggest that the correlation is significant rejecting the null hypothesis (H01) and confirming the alternative hypothesis (H1). As per Cohen ( Carson, 2012), effect size of 0.2 is small, 0.5 is medium and > 0.8 is large. The heterogeneity analysis shows a Q statistic of 1097.47 and a p value of 0.000 denoting significant heterogeneity, which is also evident from the different effect sizes as shown in Forest plot. While a few studies have reported a negative correlation, the majority of studies have reported a positive correlation with one study reporting a correlation coefficient of 0.98 (blue circle on right with no black line). The high value of I ^2^ (98.7%) again shows the very high level of heterogeneity. The large prediction interval (-0.70 to 0.95) suggests that the studies included for meta-analysis are very different from each other and it is difficult to draw any straightforward conclusions regarding significant effect sizes. The main reason for this is the limited number of studies and small sample sizes. Other reasons of heterogeneity could be socio-cultural factors, regional differences (for example North and South India), educational and financial backgrounds etc. One parameter, which was available for all studies is the type of teachers included as sample population. This was subjected to a subgroup analysis to see whether it is a significant factor, or if there is a similarity among studies with similar teacher types.

The results of subgroup analysis, as shown in Table 4, reveal two things. First, despite these subgroups being made on the presumption that there may be differences in teachers according to their level of teaching, there will be some similarity within groups. But the high degree of heterogeneity as expressed by I ^2^ (98%, 99%, 95% for groups college, school and trainee respectively) suggests that even within these subgroups, there is a lot of differences between study samples. Second, the differences between subgroups are not significant (p value of 0.823). Both of these conclusions indicate that level of teaching is not a significant factor affecting the correlation of EI with teachers’ personal health and there are other factors contributing to heterogeneity of studies.

**Table 4.** Subgroup analysis of personal health with teacher type.

Subgroup name	Correlation	CI Lower limit	CI Upper limit	Weight	Q	p _Q_	I ^2^	T ^2^	T	PI LL	PI UL
College	0.59	-1.00	1.00	12.27%	65.06	0.00	98%	0.45	0.67	-1.00	1.00
School	0.46	-0.06	0.78	30.39%	970.26	0.00	99%	0.45	0.67	-0.81	0.97
Trainee	0.30	-0.43	0.79	57.34%	37.93	0.00	95%	0.45	0.67	-0.99	1.00

Analysis of variance	Sum of squares (Q*)	df	p
Between/Model	0.39	2	0.823

### Meta-analysis of EI and professional health of teachers

**Figure 3.**
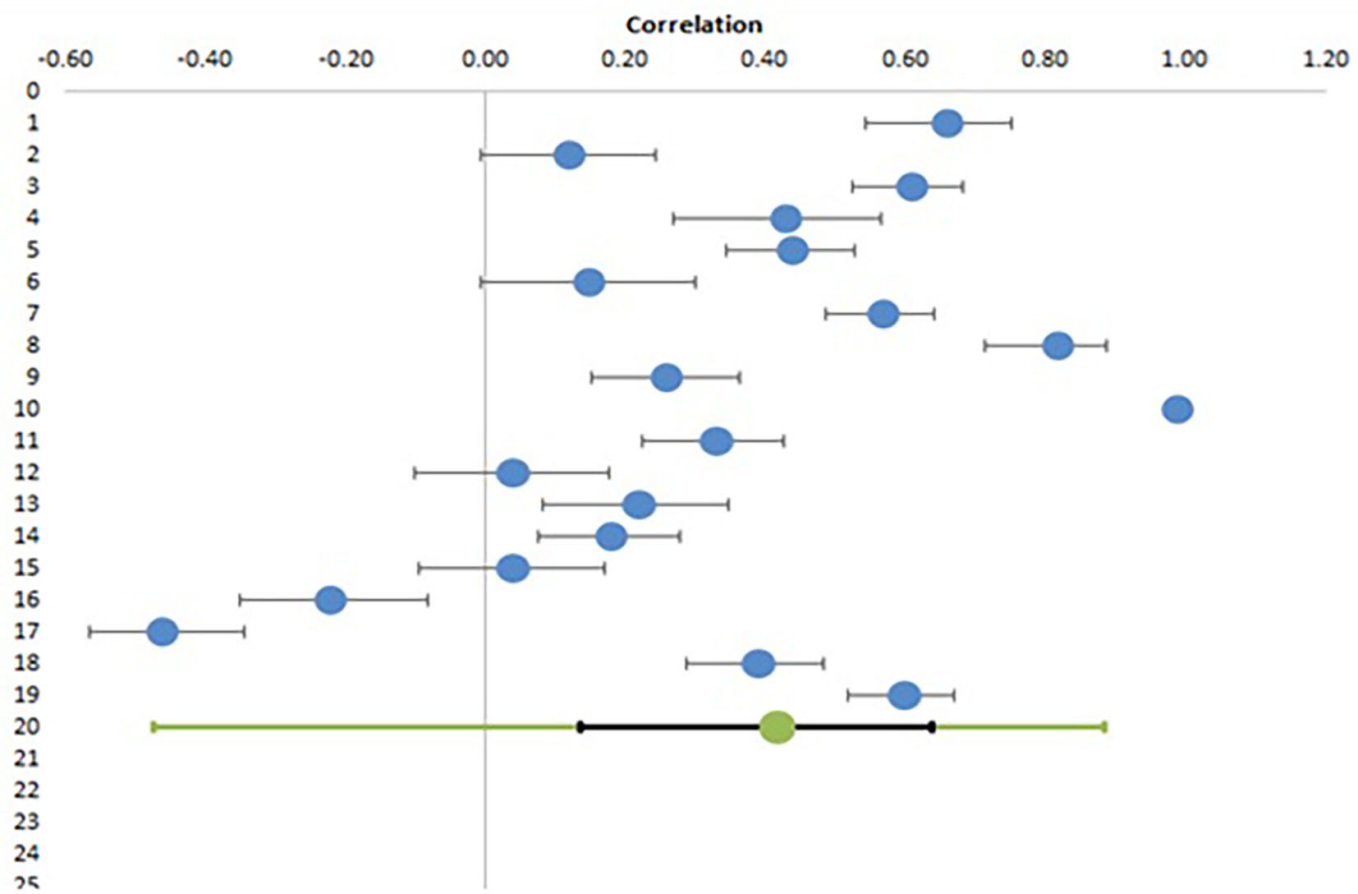
Forest plot for meta-analysis of correlation of EI with professional health.

From eighteen studies examining the relationship of EI with teachers’ professional health parameters, nineteen effect sizes among 4165 participants were deduced and included in meta-analysis. Table 5 shows the correlation coefficients of EI with parameters associated with professional health along with their confidence intervals and relative weights. Table 6 shows the combined effect size with confidence and prediction interval and heterogeneity data. The study by Anjum and Swathi (2017) reported a correlation of 1, which was changed to 0.99 for proper calculation as some deductions are not possible with this coefficient level. Figure 3 shows the Forest plot for this meta-analysis.

**Table 5.** Correlation of EI with professional health parameters.

Study name	Correlation	CI Lower limit	CI Upper limit	Weight
Lenka *et al.*, 2012	0.66	0.54	0.75	5.19%
Mandip *et al.*, 2012	0.12	-0.01	0.24	5.31%
Jha and Singh, 2012	0.61	0.53	0.68	5.31%
Choudhary and Choudhary, 2013	0.43	0.27	0.57	5.19%
Begum and Khan, 2015	0.44	0.34	0.53	5.33%
Kumar, 2016	0.15	-0.01	0.30	5.25%
Singh and Kumar, 2016	0.57	0.49	0.64	5.33%
Rani and Prasad, 2016	0.82	0.72	0.89	4.99%
Chaturvedi and Mishra, 2017	0.26	0.15	0.36	5.33%
Anjum and Swathi, 2017	0.99	0.98	0.99	4.96%
Geetha and Sripirabaa, 2017	0.33	0.22	0.43	5.33%
Garg and Islam, 2018	0.04	-0.10	0.18	5.29%
Rajam Jeya Devi and Kumar, 2018	0.22	0.08	0.35	5.29%
Soanes and Sungoh, 2019	0.18	0.08	0.28	5.35%
Mohammad, 2020	0.04	-0.09	0.17	5.30%
Kant and Shanker, 2021	-0.22	-0.35	-0.08	5.29%
Kumara, 2021	-0.46	-0.56	-0.34	5.29%
Pradhan *et al.*, 2022	0.39	0.29	0.48	5.33%
Pradhan *et al*., 2022	0.60	0.52	0.67	5.33%

**Table 6.** Meta-analysis 2 with combined effect and heterogeneity.

**Meta-analysis model**		**Number of incl. subjects**	4165
		**Number of incl. studies**	19
Model	Random effect model	Z-value	3.03
		One-tailed p-value	0.001
Confidence level	95%	Two-tailed p-value	0.002
**Combined effect size**		**Heterogeneity**	
**Correlation**	0.42	Q	741.05
Confidence interval LL	0.14	p _Q_	0.000
Confidence interval UL	0.64	I ^2^	97.57%
Prediction interval LL	-0.47	T ^2^ (z)	0.19
Prediction interval UL	0.89	T (z)	0.43

The above analysis shows a significant correlation of EI with professional health of teachers with a combined effect size of 0.42 (95% CI, 0.14-0.64). Similar to previous results, there is a lot of heterogeneity among studies, as suggested by an I ^2^ value of 97.5%. The Z value of 3.03 and low p values (<0.05) suggest that the combined effect size is significant rejecting the null hypothesis (H01) and confirming alternative hypothesis (H1). The forest plot also depicts the significant combined effect size in the bottom line with green circle and confidence interval in black. As can be seen from this plot, the difference among the results of studies is very marked, such that while some studies show a negative correlation, others have reported very high positive correlation with one study reporting it as 0.99 (blue circle on extreme right without a black line). This again makes the prediction interval very large (-0.47 – 0.89), making the statistical significance of combined effect size less strong.

For subgroup analysis, only two groups were defined (school and college) as there was only one study with trainee teachers.

As shown in Table 7, there is still a very high degree of heterogeneity within these subgroups (I ^2^ of 97% and 98% in college and school subgroup) and there is no significant difference in pooled effect sizes between groups (p value of 0.169). One interesting aspect of these results is that while for personal health parameters, the effect size is more for college teachers as compared to school teachers, the reverse is true for professional health parameters. The higher Q (1.89 versus 0.39) with lower p value (0.169 versus 0.823), although not statistically significant, may suggest a trend which can be explored in future studies with larger sample size.

**Table 7.** Subgroup analysis of professional health with teacher type.

Subgroup name	Correlation	CI Lower limit	CI Upper limit	Weight	Q	p _Q_	I ^2^	T ^2^	T	PI LL	PI UL
College	0.30	-0.03	0.57	80.06%	323.85	0.00	97%	0.21	0.46	-0.66	0.89
School	0.55	-0.09	0.87	19.94%	400.85	0.00	98%	0.21	0.46	-0.60	0.96

Analysis of variance	Sum of squares (Q*)	df	p
Between/Model	1.89	1	0.169

The general inference which can be drawn from both these meta-analyses of available research is that although teachers’ EI is significantly correlated with both personal and professional health parameters, the high level of heterogeneity resulting in a large prediction interval suggests that larger studies with bigger sample size are still needed so that the strength of association and significance of results can have a more meaningful interpretation.

### Meta-analysis of EI and gender

**Figure 4.**
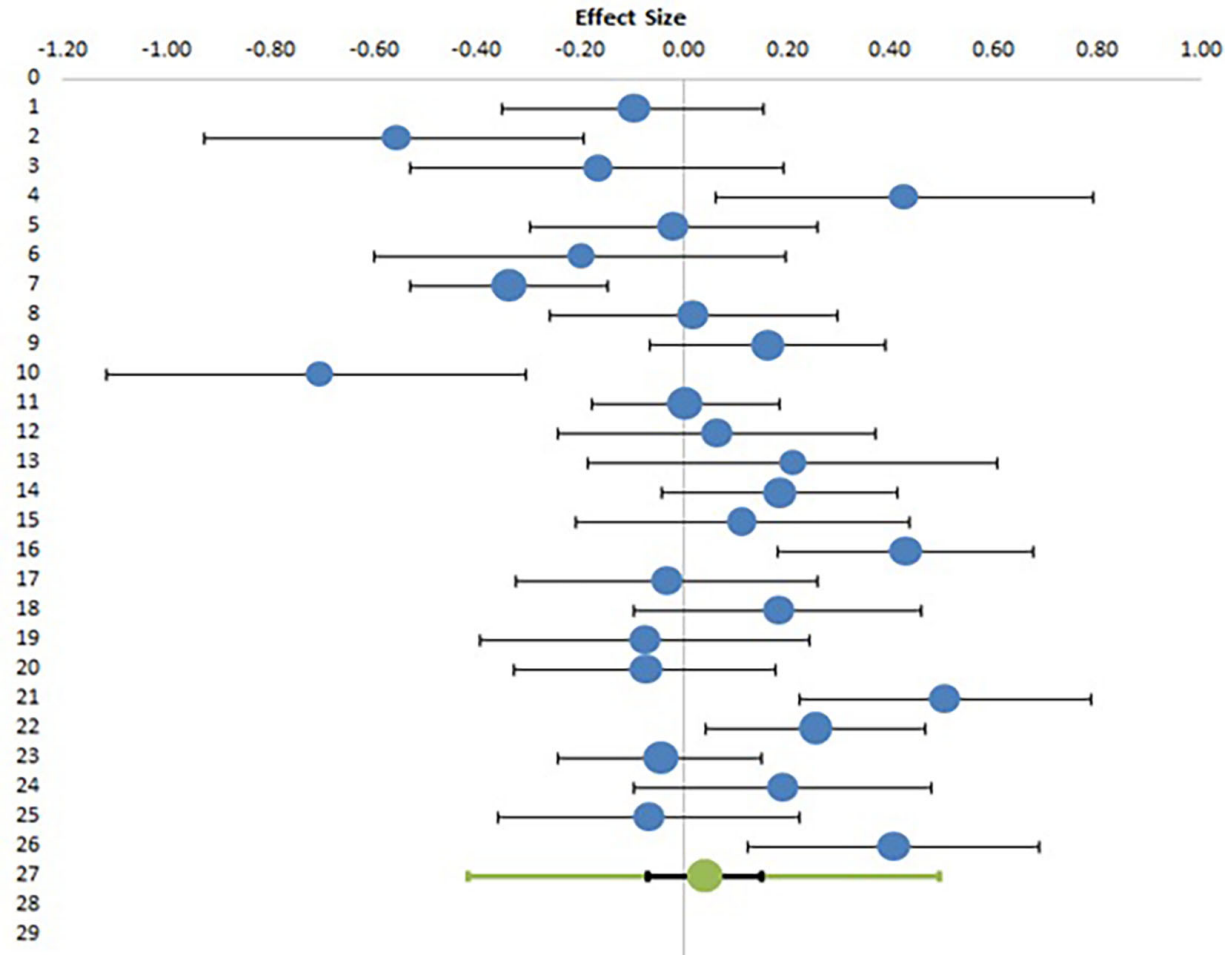
Forest plot of meta-analysis of gender effect on EI.

Studies evaluating the effect of gender on EI have reported conflicting results, with five studies reporting higher EI in female teachers as compared to males, five studies reporting higher EI in males as compared to females and sixteen studies reporting no effect of gender on EI, while twenty-nine studies did not specifically look into this relationship. Six studies evaluated the effect of gender on EI, but had inadequate data. Finally, twenty-six studies with appropriate datasets were included in meta-analysis involving 6005 participants. Table 8 presents the summary statistics and calculation of effect size in the form of Hedges’ g with confidence interval and relative weights. Only means, standard deviations and sample size were used for calculation of Hedges’ g, t values are extracted from studies but not used for this calculation. Table 9 shows the result of meta-analysis with combined effect size with confidence intervals, along with heterogeneity data. Figure 4 shows the Forest plot for the meta-analysis. For all parameters, first column (1) denotes values for males and second column (2) denotes values for females.

**Table 8.** Summary statistics and effect size calculation for studies included for meta-analysis of gender effect on EI.

Study name	M _1_	M _2_	S _1_	S _2_	n _1_	n _2_	t-value	Hedge’s g	CI-LL	CI-UL	Weights
Jha and Singh, 2012	139.1	138.2	9.19	9.26	147	103	0.445	-0.10	-0.35	0.15	4.13%
Lenka *et al.*, 2012	136.2	128.2	15.98	12.04	60	60	3.04	-0.56	-0.93	-0.19	3.21%
Mishra and Laskar, 2012	757.8	742.8	84.5	94	60	60	0.92	-0.17	-0.53	0.19	3.26%
Choudhary and Choudhary, 2013	110.2	116.9	16.4	14.8	60	60	2.37	0.42	0.06	0.79	3.24%
Kant and Lenka, 2013	137.2	136.9	14.55	9.65	100	100	0.074	-0.02	-0.30	0.26	3.92%
Nisha and Budhisagar, 2013	137.2	133.6	15.78	20.22	45	55	0.997	-0.20	-0.60	0.20	3.00%
Surana and Rawat, 2014	68.22	64.31	11.47	11.62	166	311	3.515	-0.34	-0.53	-0.15	4.66%
Kant, 2014	17.98	18.15	11.64	5.25	100	100	0.56	0.02	-0.26	0.30	3.92%
Begum and Khan, 2015	66.1	67.6	9.8	8.7	150	150	1.97	0.16	-0.07	0.39	4.35%
Maheshwari and Balamulu, 2015	31.75	30.05	2.55	2.23	50	50	3.59	-0.70	-1.12	-0.30	2.93%
Charankumar, 2015	139	139.1	15.39	15.19	303	189	0.03	0.00	-0.18	0.19	4.73%
Mehta 2015	3.65	3.69	0.65	0.62	49	251	0.35	0.06	-0.24	0.37	3.67%
Das, 2016	79.14	81.61	10.25	12.96	50	50	1.05	0.21	-0.18	0.61	3.01%
Chaturvedi and Mishra, 2017	117.6	120.9	16.44	18.6	150	150	2.64	0.18	-0.04	0.41	4.35%
Kaur, 2017	677.3	685.4	73.44	69.33	75	75	0.489	0.11	-0.21	0.43	3.56%
Reddy, 2017	164.4	183.3	40.64	52.59	244	88	3.45	0.43	0.18	0.68	4.18%
Garg and Islam, 2018	69.13	68.96	5.42	5.15	70	130	0.215	-0.03	-0.32	0.26	3.80%
Singh and Singh, 2018	114	116.7	15.03	13.98	100	100	0.7	0.18	-0.10	0.46	3.91%
Suresh and Srinivasan, 2018	29.52	29.04	6.52	6.09	79	73	0.466	-0.08	-0.40	0.24	3.58%
Toor and Kang, 2018	130.5	129.2	17.02	18.37	120	120	0.572	-0.07	-0.33	0.18	4.12%
Pugazhenthi and Srinivasan, 2018	59.99	64.68	9.68	8.79	100	100	3.6	0.51	0.23	0.79	3.88%
Soanes and Sungoh, 2019	777.4	800.3	94.54	85.94	144	208	2.36	0.25	0.04	0.47	4.46%
Sandhu and Agrawal, 2020	220.3	219.3	21.44	21.32	214	186	2.08	-0.05	-0.24	0.15	4.61%
Kamboj and Garg, 2021	94.29	96.61	14.14	10.69	77	123	1.32	0.19	-0.09	0.48	3.85%
Kant and Shanker, 2021	116.4	113.5	46.94	35.6	130	70	0.491	-0.07	-0.36	0.22	3.80%
Kumara, 2021	128.9	132.5	10.65	5.91	110	90	0.657	0.41	0.13	0.69	3.88%

**Table 9.** Meta-analysis with combined effect size and heterogeneity.

**Meta-analysis model**		**Number of incl. subjects**	6005
		**Number of incl. studies**	26
Model	Random effect model	Z-value	0.74
		One-tailed p-value	0.231
Confidence level	95%	Two-tailed p-value	0.461
**Combined effect size**		**Heterogeneity**	
**Hedges’ g (SE)**	**0.04 (SE 0.05)**	Q	86.67
Confidence interval LL	-0.07	p _Q_	0.000
Confidence interval UL	0.15	I ^2^	71.16%
Prediction interval LL	-0.41	T ^2^ (z)	0.05
Prediction interval UL	0.49	T (z)	0.21

The above meta-analysis clearly shows that the combined effect size, or the difference between male and female teachers’ EI scores is negligible (0.04) which is too small an effect to be either statistically significant (p value more than 0.05) or have any practical significance. The Forest plot, as depicted in Figure 4 shows that with the exception of a few studies showing a strong effect on either direction, the majority of studies have reported a very small difference among genders. This result confirms our second null hypothesis (H02) and rejects alternative hypothesis (H2) and concludes that there is no significant difference between the emotional intelligence of male and female teachers. The heterogeneity data shows an I ^2^ value of 71%, which, although high, is much lower than the previous two meta-analyses.

### Publication bias analysis

**Figure 5.**
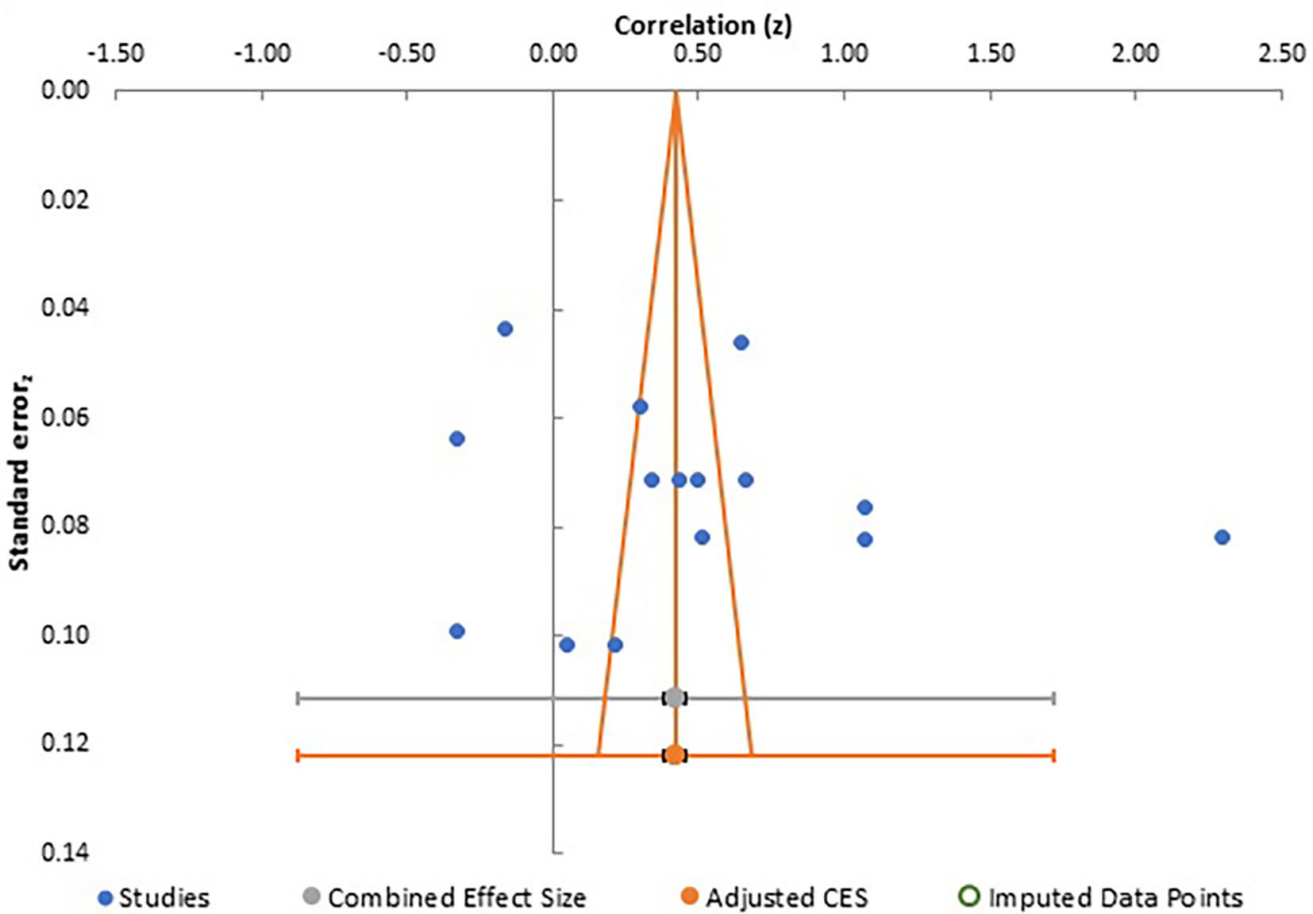
Funnel plot for meta-analysis 1.

**Figure 6.**
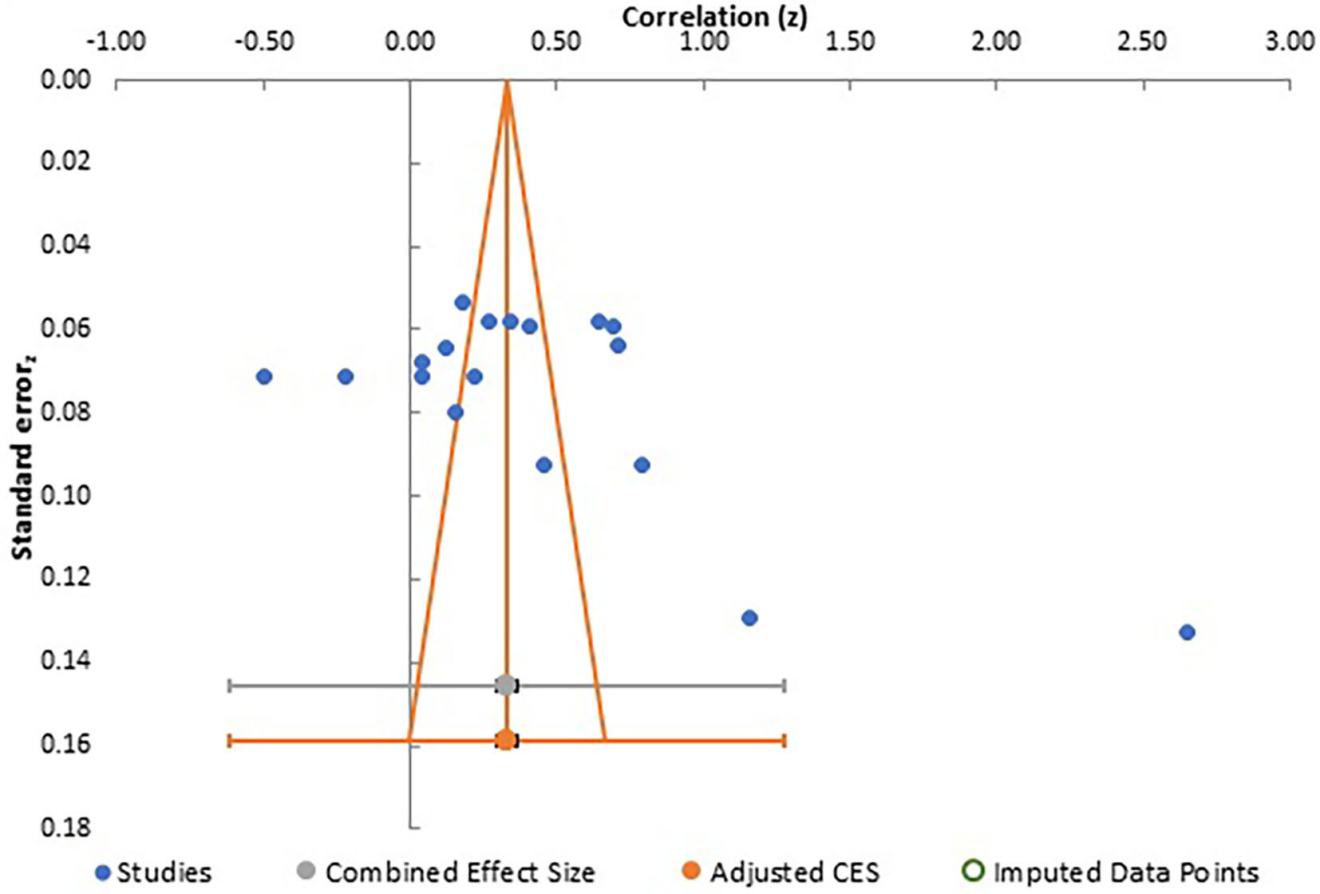
Funnel plot for meta-analysis 2.

**Figure 7.**
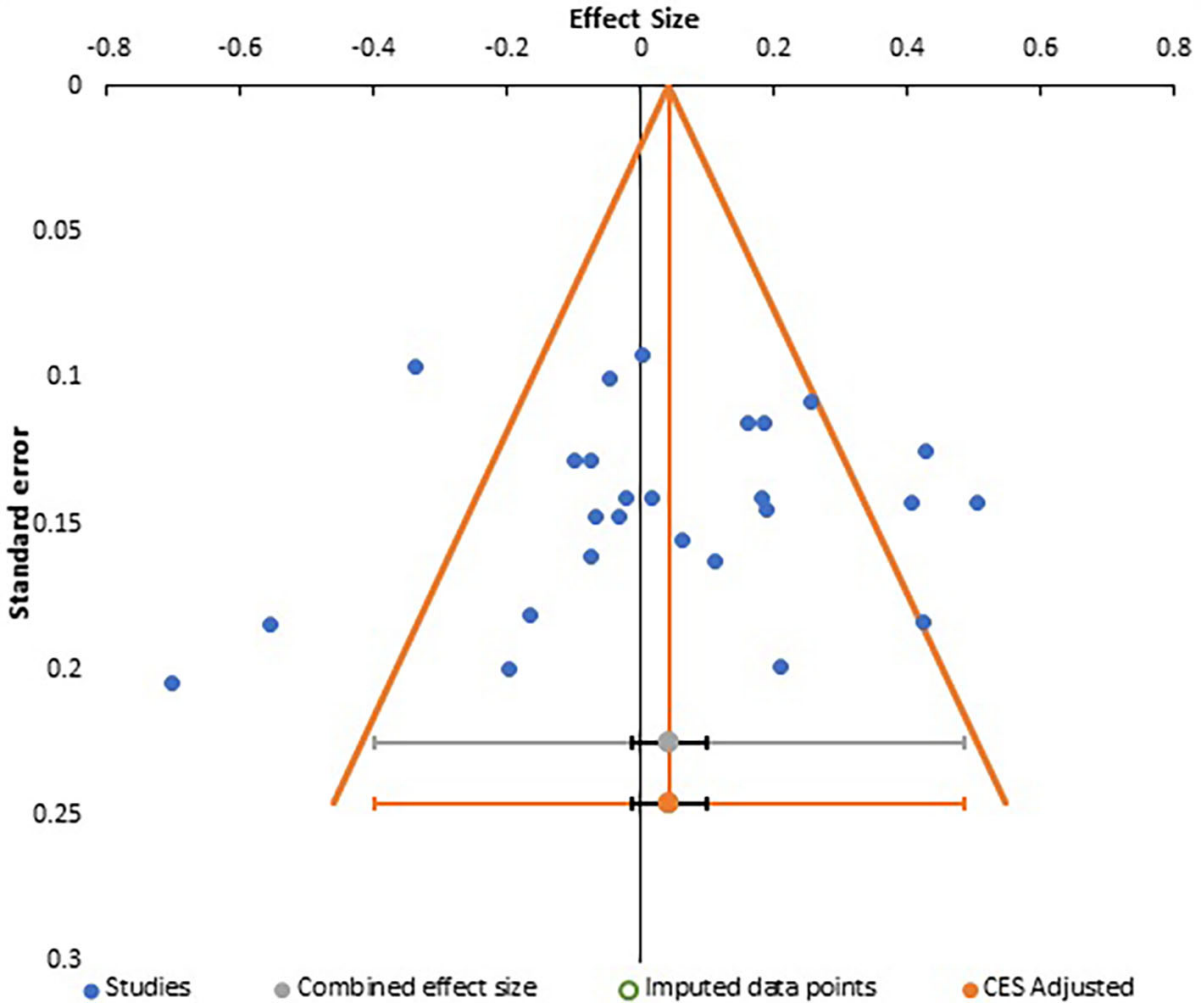
Funnel plot for meta-analysis 3.

Publication bias analysis for all three meta-analyses are summarized together with a depiction of funnel plots in Figures 5, 6 and 7 with Egger regression analysis in Tables 10, 11 and 12.

**Table 10.** Egger regression for meta-analysis 1.

Egger regression				
	Estimate	SE	CI LL	CI UL
Intercept	8.69	8.93	-10.46	27.83
Slope	-0.15	0.61	-1.45	1.15

t test	0.97
p-value	0.35

**Table 11.** Egger regression for meta-analysis 1.

Egger regression				
	Estimate	SE	CI LL	CI UL
Intercept	12.42	6.71	-1.75	26.59
Slope	-0.50	0.46	-1.48	0.47

t test	1.85
p-value	0.08

**Table 12.** Egger regression for meta-analysis 3.

Egger regression				
	Estimate	Standard error	CI LL	CI UL
Intercept	-0.51	1.70	-4.01	2.99
Slope	0.11	0.23	-0.36	0.59

t test	-0.30
p-value	0.766

Careful analysis of these funnel plots suggests that, although there was no statistically significant bias in all three meta-analysis, as there is no linear relationship between the x and y axes and effect sizes are almost equally distributed on both sides of the combined effect size in a near funnel shape, there is still some bias present in all three. This is obviously more apparent in the first two plots with presence of a single study with very strong positive correlation, which somewhat skews the shape of the funnel. The same conclusion can be drawn from analysis of Egger’s intercept which is highest in the second meta-analysis and least (near zero) in the third meta-analysis. Although the p-value is insignificant in all three, the near perfect funnel shape and lowest value of Egger’s intercept make the third meta-analysis least affected by publication bias. The obvious reasons are larger number of studies in this analysis. Therefore, the general inference which can be drawn from study of publication bias is that the third meta-analysis, studying the effect of gender presents the most reliable data in terms of publication bias.

### Sensitivity analysis

For all three meta-analyses, two alterations were planned namely in sample size and effect size. In the first analysis, studies with a sample size of 100 or less and then less than 200 were removed. For effect size alteration, in the first two meta-analysis, studies with effect size greater than 0.9 and then greater than 0.75 were removed. In the third meta-analysis, this was reduced to effect size greater than 0.5 after an evaluation of the results. The purpose of these alterations is to see how sensitive the final results are to these changes and what is the robustness of the results. As can be seen from Table 13, there is little or no difference in heterogeneity and p values with these changes. In the third meta-analysis, the change in effect size is also minimal. However, in the first two meta-analysis, the effect size drops to some extent with exclusion of studies with a sample size less than 200 and by exclusion of studies with greater effect sizes. As the sample size is inversely related to standard error (which can be a surrogate for the accuracy of results), the results of sensitivity analysis point to the fact that although the relationship between studied parameters is positive and significant, the pooled effect size of more accurate studies is somewhat smaller. This, coupled with a high degree of heterogeneity is further argument for designing future studies with strong methodologies. The sensitivity analysis for the third meta-analysis clearly suggests the robustness of the results, similar to the conclusion of publication bias analysis.

**Table 13.** Sensitivity analysis for all three meta-analyses.

		Combined ES (95% CI)	Heterogeneity (I ^2^)	P value
**Sensitivity analysis for 1** ^**st**^ **meta-analysis**	*Total*	*0.45 (0.12-0.69)*	*98%*	*<0.05*
	N ≤100 removed	0.49 (0.12 – 0.74)	98%	< 0.05
	N < 200 removed	0.29 ( -0.01-0.54)	97%	< 0.05
	r > 0.9 removed	0.34 (0.10-0.55)	97%	< 0.05
	r > 0.75 removed	0.23 (0.01-0.43)	96%	< 0.05
**Sensitivity analysis for 2** ^**nd**^ **meta-analysis**	*Total*	*0.42 (0.14-0.64)*	*97%*	*<0.05*
	N ≤100 removed	0.28 (0.11-0.43)	96%	< 0.05
	N < 200 removed	0.22 (0.02-0.41)	96%	< 0.05
	r > 0.9 removed	0.32 (0.14-0.48)	96%	< 0.05
	r > 0.75 removed	0.28 (0.11-0.43)	96%	< 0.05
**Sensitivity analysis for 3** ^**rd**^ **meta-analysis**	*Total*	*0.04 (-0.07-0.15)*	*71%*	*>0.05*
	N ≤100 removed	0.07 (-0.04-0.17)	68%	>0.05
	N < 200 removed	0.09 (-0.02-0.20)	68%	>0.05
	r > 0.5 removed	0.06 (-0.02- -0.15)	57%	>0.05

To see whether the heterogeneity is only because of inclusion of studies with negative effects, a tentative sensitivity analysis was done excluding such studies (not shown in the table), but did not affect the heterogeneity (93% in both), which implies that even among the studies with positive effects, there is marked heterogeneity.

### Certainty or confidence of results

Given the combination of low sample size in many studies resulting in low precision, high risk of bias due to absence or inadequate randomization, marked heterogeneity and high level of publication bias, the overall certainty of results can be labelled as low.

Table 14 summarizes the results of all three meta-analyses. Because of least amount of heterogeneity and publication bias and previously discussed sensitivity analysis, the third meta-analysis examining the effect of gender on EI has the most reliable and robust conclusion.

**Table 14.** Summary of results of all three meta-analyses.

Meta-analysis	k	N	ES	CI	I ^2^	Egger’s intercept
EI and Personal Health	15	3291	0.45	(0.12-0.69)	98	8.69
EI and Professional Health	19	4165	0.42	(0.14-0.64)	97	12.42
EI and Gender	26	6005	0.04	(-0.07-0.15)	71	-0.51

k – number of effect sizes, N – number of participants, ES – effect size, CI – confidence interval, I ^2^ – heterogeneity.

## Discussion

The aim of writing this review was to present a qualitative synthesis of the available literature on EI among Indian teachers. We have tried to include as many studies as possible, but despite that, it may be possible that some studies have been missed, especially if they are not easily accessible online. The review shows clearly that a robust data exists regarding EI in the education sector with studies done among different types of teachers with a reasonable number of participants in some of the studies. The salient features are as follows.

Maximum studies have been conducted on secondary school teachers, closely followed by trainee teachers. However, very few studies have taken participants from more than one type or level of educational institutes. Some of the studies had adequate sample size but not all studies detailed the sampling methods, demographic details, educational and other professional background and studied the relationship of these factors with EI. The most commonly utilized EI scale was the indigenous tool developed by Hyde *et al.*, followed by teacher EI inventory by Mangal. Although the majority of scholars and tool developers have not specifically mentioned this, the majority of tools are trait measures. The ease of administration and short time duration, especially in the digital age, along with better correlation with adaptive and other psychological outcomes are the main reasons for this trend. All studies using the Hyde scale have reported moderate to high level of EI scores of teachers, which needs comparative analysis with other professions with the same tool. A positive correlation with teacher effectiveness, job satisfaction, attitude, self-actualization, life satisfaction, physical and mental health, and negative correlation with occupational stress and burnout has been reported which emphasizes the role of EI in both personal and professional life of teachers. There was inadequate explanation for some of the associations like general intelligence, professional qualifications or level of teaching reported in some studies which did not discuss the findings in detail, especially given the evolving conceptual framework of trait EI which places it as a separate entity than cognitive intelligence bearing no relation with educational qualification or conventional forms of intelligence. Lack of detailed references especially regarding the measurement tool used, inadequate description of EI tool and inadequate discussion of results are some of the major limitations detected in some of the studies.

Being the first such attempt regarding Indian studies on EI, all efforts were put to include maximum number of studies to make the data as representative as possible. The exclusion was done only either in case of too small a sample size or marked deficiency in methodology or statistical analysis. Fifty-five studies were available for the qualitative analysis and three broad conclusions can be drawn from them. First, trait measures have been used by almost all researchers but the individual studies have marked heterogeneity in the scale used with inadequate information about some of the scales being used. Second, role of demographic factors (age) and professional (educational) qualification has been rarely studied and data on only the effect of gender on EI is available but with conflicting results. And finally, a lot of parameters concerning personal and professional health of teachers have been studied in relation to EI with some common features, but lacking a clear understanding and unifying inference.

Meta-analytical methods were employed subsequently to synthesize the major findings of the included studies. After evaluation of all the available data independently by both the authors, it was decided that three meta-analyses should be carried out. To address the issue of the second parameter studied in relation to EI and to cumulatively calculate combined effect size of the relationship between EI and the different parameters studied, all such parameters were grouped into two broad categories of personal and professional health parameters and then two different meta-analyses were carried out to test the hypothesis of whether EI is significantly associated with personal and professional health of teachers. Fifteen effect sizes were deduced from ten papers evaluating correlation of EI with personal health parameters and included in first meta-analysis. Nineteen effect sizes were deduced from eighteen papers evaluating correlation of EI with professional health parameters. Random effect modelling with Fisher’s r to z transformation was used to calculate combined effect size. The results of these meta-analyses show that although a significant relationship exists between EI and parameters concerned with both personal and professional health, irrespective of the level of teaching, the results are to be interpreted with the understanding that a high degree of heterogeneity exists between the studies which makes the level of significance less robust. The reasons for heterogeneity could be small sample size in many studies, inherent differences in demographic and professional parameters of sample population, different methodologies and statistical tools used and marked variations in correlation coefficients reported. One reason for apparent heterogeneity could be incorrect grouping of these parameters into personal or professional health parameters, but the more likely reason is an inherent difference in results, best exemplified by studies evaluating correlation of EI with Teacher Effectiveness. Among the seven effect sizes deduced from five papers, three were statistically insignificant while one showed a strong positive correlation. This suggests that larger studies with better methodologies are still needed to reduce such heterogeneity.

The third meta-analysis was carried out to study the effect of gender on teachers’ EI. Twenty-six papers had adequate data regarding sample size of both genders, mean EI scores and standard deviations. Hedges’ g was used as effect size. The results showed that there is no significant difference in EI between male and female teachers. The combined effect size of 0.04, coupled with a lesser degree of heterogeneity and the least amount of publication bias among the three meta-analyses implies the relatively more robust conclusion regarding lack of gender effect on EI.

Publication bias analysis was carried out by Funnel plot and Egger’s intercept regression.

The low publication bias can be attributed to two factors. First, the less stringent inclusion criteria with exclusion of limited number of studies may have resulted in data with some compromise in quality but broader inclusion results in less selection bias. As this is the first such exclusive meta-analysis of Indian studies, more emphasis was placed on inclusion of maximum number of studies. Future researchers may attempt to carry out such analysis with more stringent inclusion criteria with more strict emphasis on methodology and reporting of results. The second reason for less bias is related to a policy decision. Early attempts in the field of meta-analysis included unpublished dissertations to reduce bias. In India, the University Grants Commission (UGC) has made it mandatory to publish papers related to one’s research work in journals as part of thesis submission before awarding doctorate degrees. Thus, looking for unpublished dissertations in thesis repositories is mostly not productive as all the theses will have an accompanying paper which must have been published in a journal.

The results of sensitivity analysis mirror the findings of publication bias analysis. Even by excluding studies with lesser participants or higher effect size, the results of the third meta-analysis remain unchanged, while there was a small change detected in first two meta-analyses.

Lack of detailed description on sampling method used, low level of randomization and lack of description of randomization methods are some of the biggest limitations in these studies which result in high risk of bias, imprecision and low certainty of evidence derived from the review.

### Future directions

One of the obvious areas of future research is designing studies with larger sample size and clearly defined tools and methodologies. The relationship of age, educational level and other professional qualification with EI needs further research. An attempt can be made to do a comparative study of an Indian scale with some internationally recognized EI measure which can help to give it an international recognition and acceptance. Pre-defined parameters for teachers’ health and performance and their correlation with EI in larger and possibly longitudinal studies will further consolidate the EI research domain.

### Limitations

High level of heterogeneity reduces the strength of conclusions from any meta-analysis. More elaborate delineations of various demographic and professional parameters are needed in future studies to analyse different subgroups to reduce this. Lesser stringent criteria for inclusion in review may have resulted in some compromise with quality of data, but care was taken to include only those studies in meta-analysis which reported all relevant metrics. Elaborate discussion regarding tools and results in each study would have improved the qualitative results of the review but every attempt was made to synthesize as much information as possible.

## Conclusion

Intelligent use of emotions is always desirable, whether in a student, teacher, corporate executive or home-maker. The usefulness of determining the role of demographic, academic and psycho-social factors in determining emotional intelligence and its impact on the health and performance of individuals goes beyond any one sector, and can help administrators and policy makers in improving the work environment, productivity and performance by adequate training programs and by specific target interventions for those who require special attention. The present work summarizes the major findings from studies done among Indian teachers and concludes that emotional intelligence positively impacts the personal and professional life of teachers. There is no difference between genders regarding trait EI measures. Longitudinal studies examining the impact of any intervention to improve EI are needed to support the argument that such interventions must be made part of routine teacher training programs which are already in place. This review can also serve as a useful baseline data for future researchers in EI domain, both in India and abroad.

## Data Availability

Figshare: Data for systematic review and meta-analysis. https://doi.org/10.6084/m9.figshare.24212460.v2 ( Pandey, 2023). This project contains the following underlying data:
- Data-total.xlsx (List of all studies with relevant details for statistical analysis) This project contains the following extended data:
- PRISMA checklist for abstract and review (2020).docx - Fig -1 - prisma flowchart for study inclusion.docx Data are available under the terms of the Creative Commons Zero “No rights reserved” data waiver (CC0 1.0 Public domain dedication).
