# Supplementary material for: Research on emotional intelligence among Indian teachers: A Systematic Review and meta-analysis of its correlation with health parameters and impact of gender
Source: F1000Res. 2024 Feb 12;12:1519. Originally published 2023 Nov 28. [Version 2] doi: 10.12688/f1000research.143151.2 (PMC10831234; doi:10.12688/f1000research.143151.2)
Supplement: Peer review history

# Reviewer report: Reviewer response for version 1

**DOI:** 10.5256/f1000research.156782.r228963

**Published:** 2024-01-31

**Authors:** Ravi Kant

**Affiliations:** 1 Central University of South Bihar,, Gaya, Bihar, India

This paper is a good attempt to explore the status of research on emotional intelligence in India. The following are some points suggested to the author-

 1. The paper is too long in comparison to the normal length of the research paper. 

 2. The rationale behind choosing the Indian research only, needs a more logical background.

 3. Methodology is sufficient and clear for a layman.

 4. I am unable to understand why researchers used two different methods in a single paper i.e. meta-analysis and survey. 

 5. References are relevant to the present study.

Are the rationale for, and objectives of, the Systematic Review clearly stated?

Yes

Is the statistical analysis and its interpretation appropriate?

Yes

Are sufficient details of the methods and analysis provided to allow replication by others?

Yes

Are the conclusions drawn adequately supported by the results presented in the review?

Yes

Reviewer Expertise:

ICT in Education, Educational Psychology, Eductional Technology, Internet Behaviour

I confirm that I have read this submission and believe that I have an appropriate level of expertise to confirm that it is of an acceptable scientific standard.

# Reviewer report: Reviewer response for version 1

**DOI:** 10.5256/f1000research.156782.r228959

**Published:** 2024-01-24

**Authors:** Monu Bhargava

**Affiliations:** 1 Vivekanand Global University, Jaipur, Rajasthan, India

The study is interesting. The research paper is well-written and structured appropriately. Objectives and discussion are matched.

 Research gap is mentioned clearly.

 However, in my opinion, the research paper should

 Although the use of PRISMA technique has been mentioned in the objective part, it has not been appropriately projected in the paper. therefore,  PRISMA flowchart can be included to explain the methodology and to reduce textual information.

 Can add a test to measure "the degree of inconsistency.

 Hypothesis testing results should be clearly explained in a separate section: hypothesis testing results.

 Grammatical and spelling mistakes must be taken care of.

Are the rationale for, and objectives of, the Systematic Review clearly stated?

Yes

Is the statistical analysis and its interpretation appropriate?

Yes

Are sufficient details of the methods and analysis provided to allow replication by others?

Yes

Are the conclusions drawn adequately supported by the results presented in the review?

Yes

Reviewer Expertise:

Financial Literacy, research methodology, HR, Banking

I confirm that I have read this submission and believe that I have an appropriate level of expertise to confirm that it is of an acceptable scientific standard.
